# Characterization of Vaginal Microbiota Associated with Pregnancy Outcomes of Artificial Insemination in Dairy Cows

**DOI:** 10.4014/jmb.2002.02010

**Published:** 2020-03-27

**Authors:** Shi-Yi Chen, Feilong Deng, Ming Zhang, Xianbo Jia, Song-Jia Lai

**Affiliations:** 1 Farm Animal Genetic Resources Exploration and Innovation Key Laboratory of Sichuan Province, Sichuan Agricultural University, Chengdu, P.R. China; 2 College of Animal Science & Technology, Sichuan Agricultural University, Chengdu, P.R. China; 3 Special Key Laboratory of Microbial Resources and Drug Development, Research Center for Medicine and Biology, Zunyi Medical University, Zunyi, P.R. China

**Keywords:** Vaginal microbiota, pregnancy, artificial insemination, 16S rRNA gene

## Abstract

The profitability of the dairy and beef industries is largely affected by the actually achieved reproductive efficiency. Although a large proportion of cows worldwide are bred by artificial insemination (AI) services, many potential factors affecting the outcome of pregnancy by AI remain to be addressed. In the present study, we investigated the vaginal microbiota by high-throughput sequencing of 16S rRNA gene and analyzed their association with differential pregnancy outcomes (*i.e*., pregnant vs. nonpregnant) of multiple AI services in dairy cows. Sequencing of the V3-V4 region totally produced 512,046 high-quality sequences that were computationally clustered into 2,584 operational taxonomic units (OTUs). All OTUs were taxonomically assigned to 10 bacterial phyla. There were statistically significant differences among the three AI service times (T1, T2 and T3) with respect to the Shannon index and number of observed OTUs (*p* < 0.05). Bray-Curtis distance-based PCoA analysis also revealed that T2 group could be significantly distinguished from T1 and T3. However, no significant difference between the pregnant and nonpregnant cows was found in confidence regarding both alpha diversity and beta diversity. These results could help us better understand the possible influence of vaginal microbial community on pregnancy outcomes of AI service in cows.

## Introduction

In contrast to polytocous domestic mammals, such as pigs and rabbits, bovines have a very low reproductive efficiency because of a long gestation period and one offspring per birth (the twinning rates of ~1% in beef and ~4% in dairy cattle) [1]. Therefore the profitability of the dairy and beef industries, especially in the intensive production farms, has been largely affected by their achieved reproductive efficiencies [2]. Currently, a considerable proportion of cows, or roughly 80% of dairy cattle in the United States, 75% in Canada and China, 35% in India and 20% in Brazil, are bred by artificial insemination (AI) service, which has a history of more than 100 years in terms of technological possibility and 50 years of commercial operation [3-6]. Despite the extremely wide applications of AI technology, there are a dozen known and unknown factors associated with the served cows that could directly or indirectly affect the pregnancy outcomes of AI service [7]. Burns and colleagues (2010) comprehensively reviewed the physiological, environmental, genetic, and infectious factors affecting reproductive efficiency in cattle [8].

It has been well acknowledged that diseased cows, either preceding by many weeks or close to insemination, especially for the occurrence of reproductive tract and uterine diseases, could significantly decrease the success rate of AI services [9]. Rodrigues *et al*. (2015) investigated vaginal microbial communities and found that they were associated with the occurrence of reproductive tract diseases [10]. Similarly, vaginal microbial communities were compared between the synchronized heifers with positive or negative clinical vaginitis [11]. Meanwhile, maintaining immunological and physiological homeostasis in both the reproductive tract and uterine is highly essential for establishing successful conception in cows [12]. In humans, it was suggested that women with abnormal vaginal microbiota were less likely to have successful early pregnancy development after in vitro fertilization treatment [13]. Because of their close relationship with disease onset and dynamic homeostasis, the resident microbial communities in the reproductive tract and uterine are speculated to positively influence reproductive ability. As far as we know, however, less is known about whether the microbial communities in the reproductive tract could also affect the success rate of AI services in clinically healthy cows. Very recently, there were two publications intended to address this topic. Ault *et al*. (2019) studied both uterine and vaginal microbial community composition prior to AI service, and found that uterine samples from nonpregnant and pregnant cows could be separately clustered significantly [14]. A similar study examined the vaginal and fecal microbial communities and their association with the pregnancy status in beef heifers [15].

In the present study, we investigated vaginal microbial community composition in healthy dairy cows using the high-throughput sequencing approach and analyzed the association with pregnancy outcomes of multiple AI services. These results may provide insights about the potential relationship between vaginal microbiota and reproductive ability in cows.

## Materials and Methods

### Ethics Statement

Study purpose, sample collection and experimental procedures involved in the present study were approved by the Institutional Animal Care and Use Committee of Sichuan Agricultural University (201718GJHZ). The animal management, AI service and sample collection was conducted at a 1,000-cow, modern dairy farm in Sichuan Province, China.

### Animals and Study Design

**Fig. 1:**
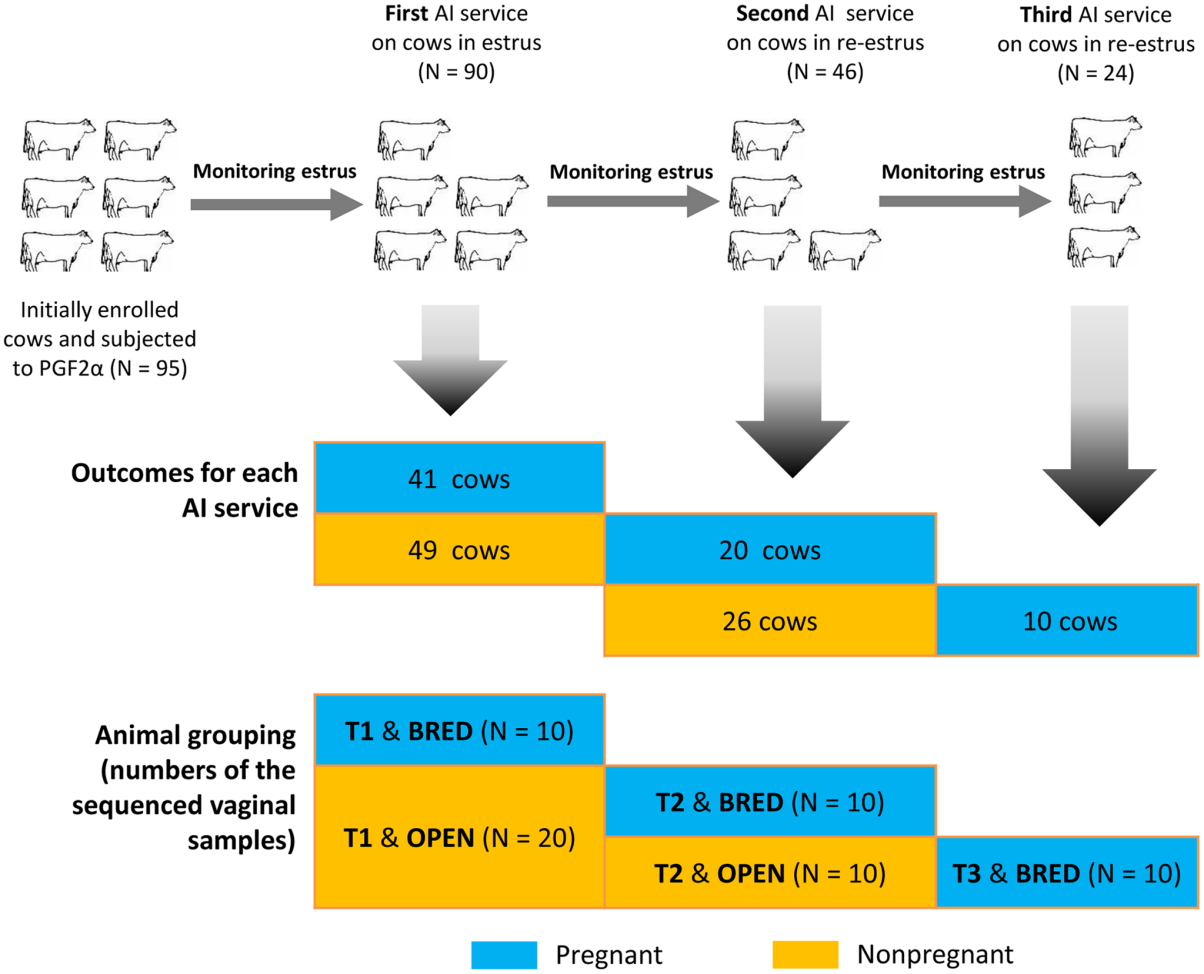
Experimental design, animal enrollment and sample collection.

The overall experimental design and cow numbers were shown in Fig. 1. A total of 95 clinically healthy and second-parity Holstein cows were initially enrolled in the present study, which had an average of 50 ± 4.5 days postpartum. All cows were housed in freestall barns with free access to fresh water and fed the same diet (all had the comparable body condition scores of 2.5~3.0). The diet formulation was shown in Table S1. Additionally, no other physiological heterogeneity was obviously observed.

All cows were first injected with 25 mg PGF2α (Dinuo, China) as a presynchronization step, and the second 25 mg PGF2α was administered with an interval of 14 days. Subsequently, visual estrus signs were monitored twice daily (at both 7:00 a.m. and 9:00 p.m., each for 20 min) until the fifth day after the second administration of PGF2α. Ninety cows had clear estrus signs and successfully subjected to the first AI service. Around 20 days after the former AI service, all re-estrus cows were resubjected to the second (N = 46) and third (N = 24) AI services, respectively. The pregnant candidates were diagnosed by ultrasound method at 35 days and 60 days after AI service, respectively. Finally, a total of 71 cows successfully conceived after the three repeated AI services. All cows falsely recognized to be pregnant or clinically diagnosed with disease by veterinarians were excluded from this study. A total of 60 vaginal samples (Fig. 1), including 30 pregnant (T1, T2, and T3) and 30 nonpregnant (T1 and T2) cows were finally selected for high-throughput sequencing.

### Vaginal Sample Collection and DNA Extraction

Vaginal swabs were ordinarily collected by double-guarded instruments according to manufacturer’s recommendation. Briefly, we carefully inserted each instrument into the vagina and upon the posterior wall of the cervix. Each swab was pushed out of the inner guard and then gently rotated against the vaginal wall for enrichment with vaginal fluids. About 15 sec later, the swab was retracted back into the inner and outer guards in order. The cotton tip was cut off and the swab was immediately placed into 1 ml Amies Transport Medium. All swab samples were stored at -80°C for future use. Also, swab samples were collected prior to AI service to avoid microbial contamination. Swabs were subjected to bacterial DNA extraction using the QIAamp BiOstic Bacteremia DNA Kit (Qiagen, China) according to manufacturer’s protocol. The DNA concentration and quality were determined by NanoVue Plus (GE Healthcare, USA), after which two samples from the second AI service were not further subjected to library construction because of inadequate DNA concentration (< 1 ng/μl in quantity and no visible electrophoretic band).

### Library Construction and Sequencing

The V3-V4 hypervariable region of bacterial 16S rRNA gene was amplified using HOTSTAR Taq Plus Master Mix Kit (Qiagen) and the universal primers (338F: 5’-ACT CCT ACG GGA GGC AGC AG-3’ and 806R: 5’-GTG GAC TAC HVG GGT WTC TAA-3’). The PCR amplification consisted of an initial denaturation step at 95°C for 4 min and 20 cycles of 95 °C for 1 min, 56°C for 45 sec, and 72°C for 1 min, followed by an extension step at 72°C for 7 min using a Bio-Rad CFX96 thermal cycler (Bio-Rad, Hercules, USA). Each sample was independently amplified in triplicate, and then further pooled and purified using a QIAquick PCR Purification Kit (Qiagen). Amplicons with both a total amount of ≥ 3 μg and OD260/280 ratio ≥ 1.8 were used to prepare sequencing libraries using an Illumina DNA Sample Preparation Kit (Illumina, USA) according to manufacturer’s instructions. Finally, libraries were sequenced on Illumina HiSeq 2000 platform for generating 300 bp paired-end reads.

### Sequence and Statistical Analysis

Paired-end reads were first merged using the “merge_pairs” function in QIIME2 with default parameters [16]. The low-quality sequences were removed by a sliding window approach with the window size of 5 bp and average Qscore < 30. The quality-filtered sequences were then processed with Deblur pipeline [17], including the removing of chimeric sequences and clustering of operational taxonomic units (OTUs) with 100% sequence similarity. The naïve Bayes classification module that was trained with Greengenes database with 99% similarity was used to annotate the representative sequences of OTUs [18]. To avoid sequencing depth bias, the number of sequences was also subsampled.

The relative abundances of OTUs, alpha diversity and beta diversity were calculated for different groups. The Kruskal-Wallis test [19] was used to detect whether statistical differences were present among multiple groups with respect to alpha diversity. The analysis of similarities (ANOSIM) was performed for testing hypotheses about the inter-group resemblances with respect to beta diversity [20]. The linear discriminant analysis (LDA) effect size (LEfSe) method was applied to detect any bacterial taxon having significantly differential abundances between groups [21]. For all statistical tests, the significance level was set as *p*-value < 0.05.

## Results

We sequenced the V3-V4 hypervariable region of 16S rRNA gene to survey vaginal microbial communities in cows. A total of 3,056,331 raw paired-end reads were generated with an average of 52,695 reads per sample (ranging from 26,981 to 99,635 reads). After the merging of overlapped paired-reads, quality filtering and removing of chimeric sequences, 512,046 high-quality sequences were finally obtained with the minimum number of 5,098 sequences per sample. According to the 100% sequence similarity, 2,584 OTUs were computationally constructed and taxonomically assigned to 10 bacterial phyla. To normalize the sequencing library sizes, all samples were subsampled to the minimum value of 5,098 sequences and then subjected to the downstream analyses.

**Fig. 2:**
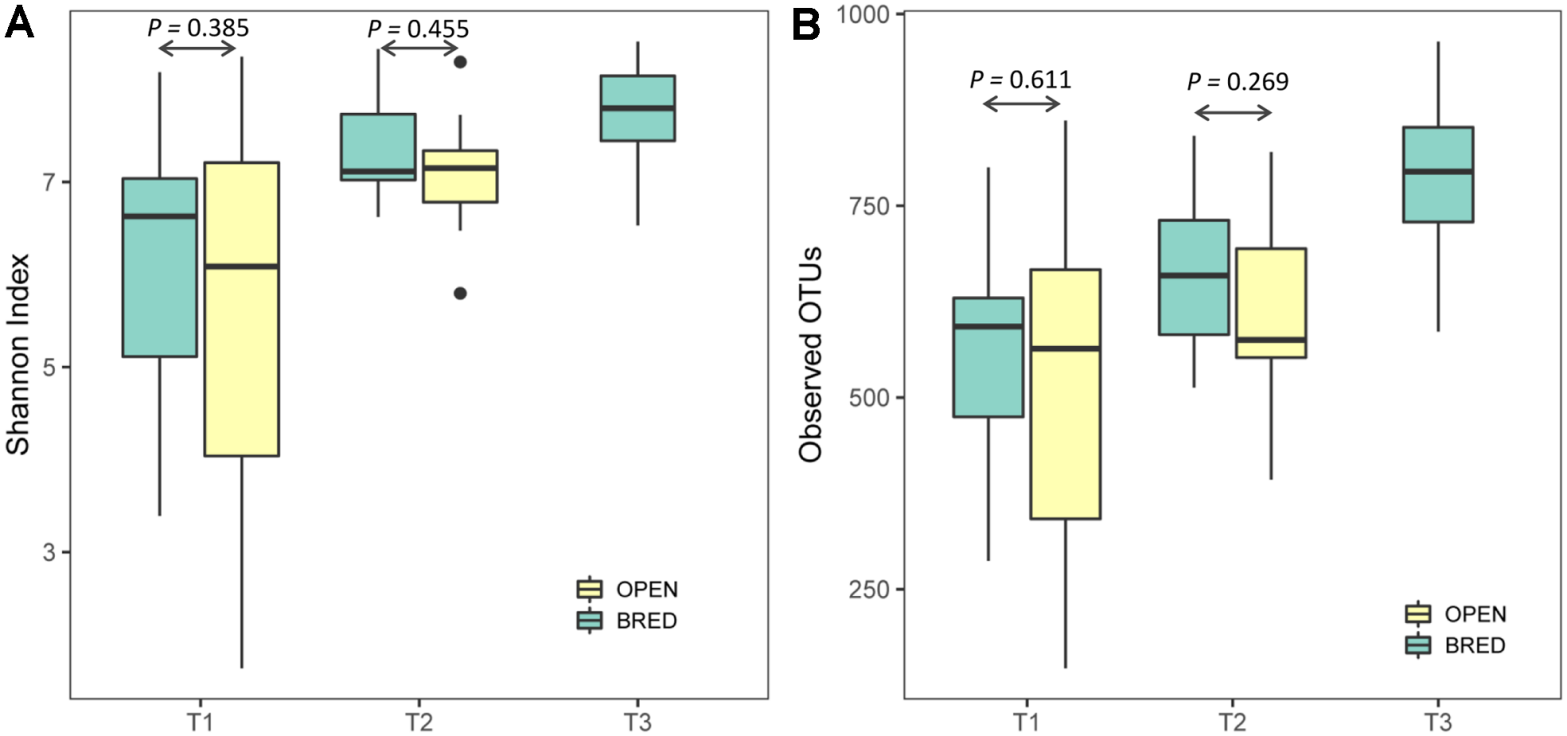
Box-plot representation of alpha diversity. Vaginal microbiota were evaluated by Shannon index (**A**) and the number of observed OTUs (**B**) among three different AI service times (T1, T2 and T3) and between pregnant (BRED) and nonpregnant (OPEN) groups.

Both species richness and diversity in the vaginal microbiota were evaluated by the number of observed OTUs and Shannon index. In comparison of the alpha diversity, there were statistically significant differences among the three AI service times (T1, T2, and T3) with respect to both the Shannon index (Fig. 2A, *P* = 0.00066 of Kruskal-Wallis test) and the number of observed OTUs (Fig. 2B, *P* = 0.00048 of Kruskal-Wallis test). However, no significant difference was found for alpha diversity between the pregnant (BRED) and nonpregnant (OPEN) groups at both T1 and T2 AI services.

**Fig. 3:**
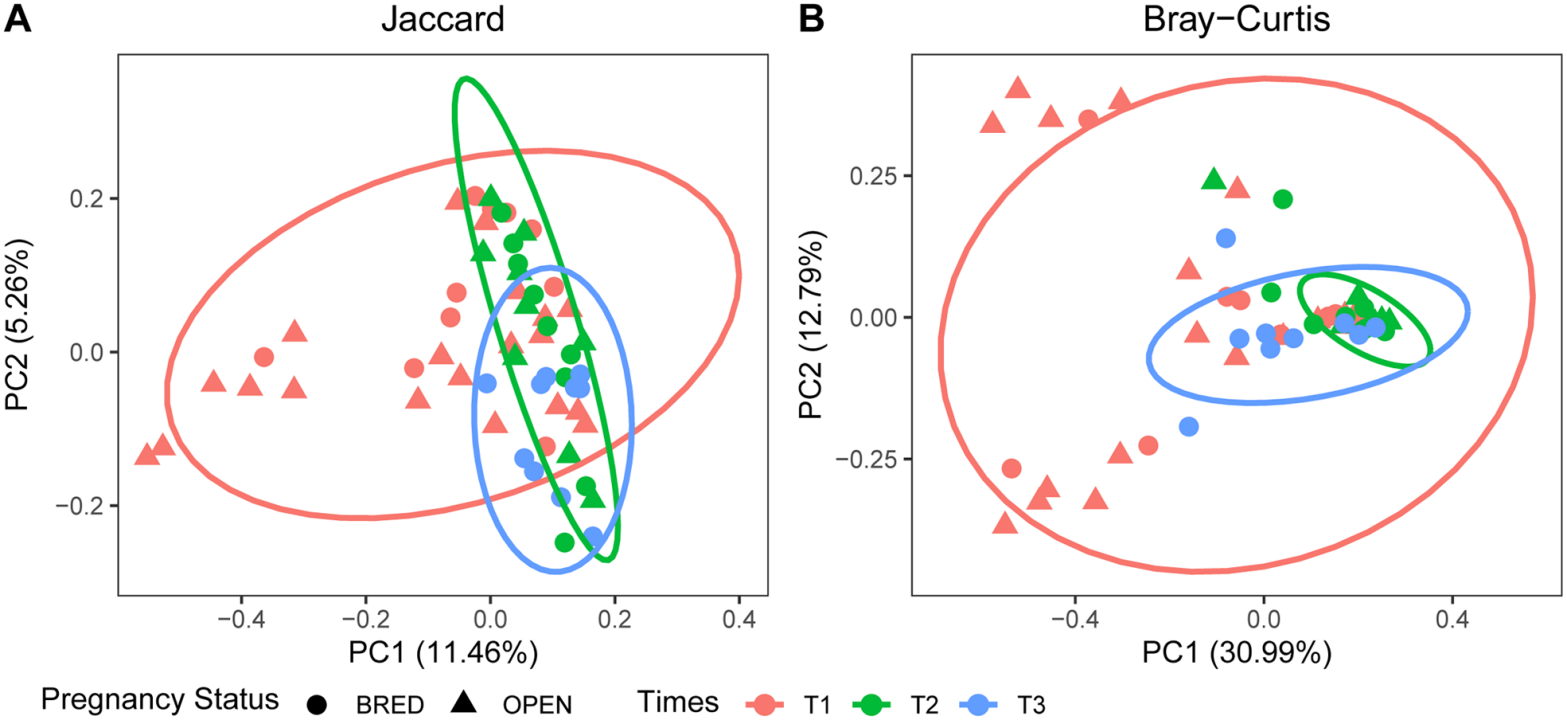
Principal Coordinates Analysis (PCoA) using Jaccard distance (A) and Bray-Curtis distance (B).

The beta diversity was further used to examine compositional dissimilarities in vaginal microbiota among the three AI service times and between the OPEN and BRED groups. The Jaccard distance-based PCoA (Principal Coordinates Analysis) showed that there was no significant difference among three AI services (Fig. 3A, ANOSIM, *p* > 0.05). In contrast, the Bray-Curtis distance-based PCoA revealed that T2 could be significantly distinguished from T1 (Fig. 3B, ANOSIM, R = 0.114, *P* = 0.038) and from T3 (Fig. 3B, ANOSIM, R = 0.283, *P* = 0.001), which indicated the compositional shift with respect to community membership and structure among different time points. However, both Jaccard and Bray-Curtis distance-based PCoA showed no significant difference between BRED and OPEN groups (Fig. 3, ANOSIM, *p* > 0.05).

**Fig. 4:**
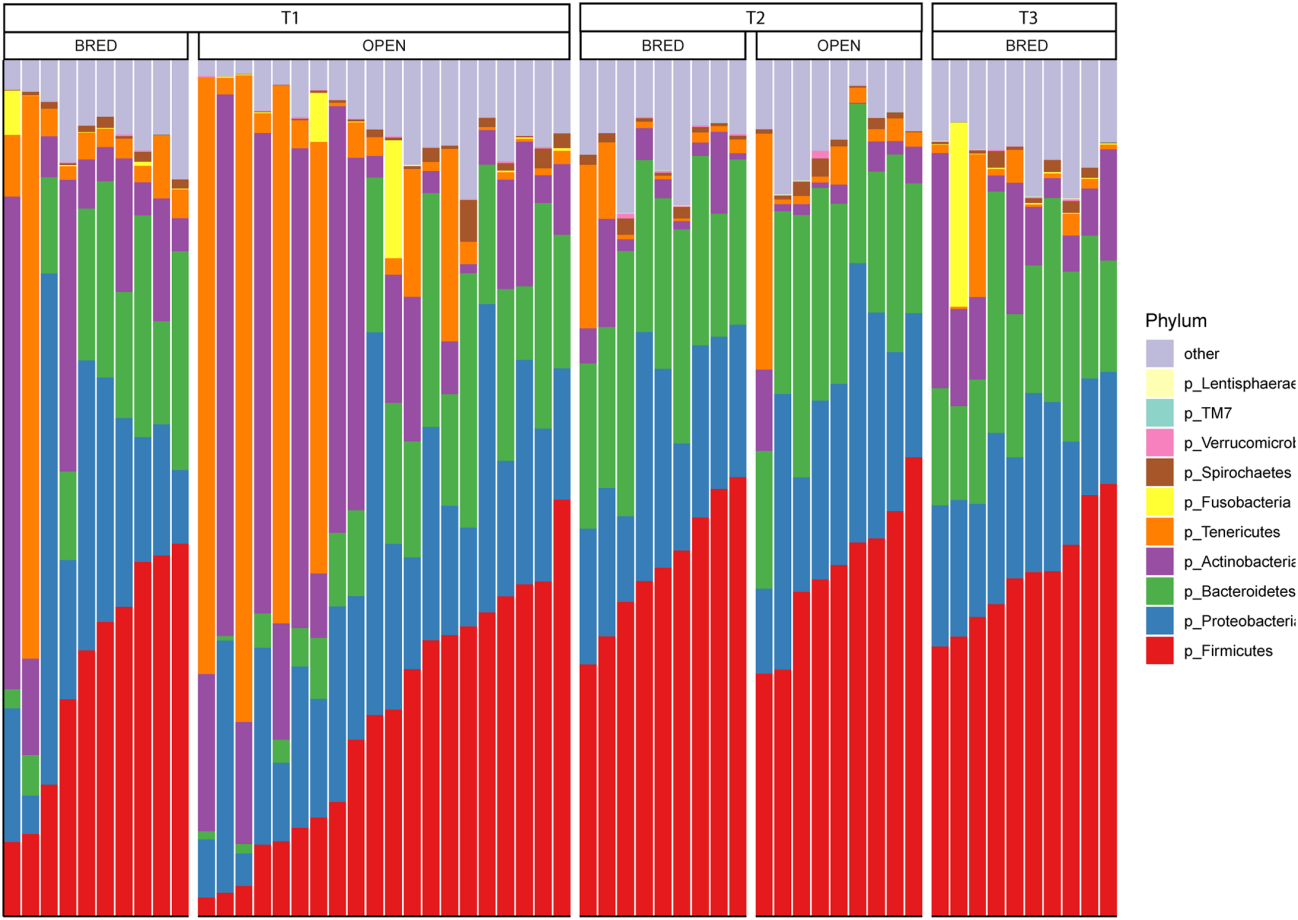
Relative abundance of vaginal microbiota at the phylum level.

**Fig. 5:**
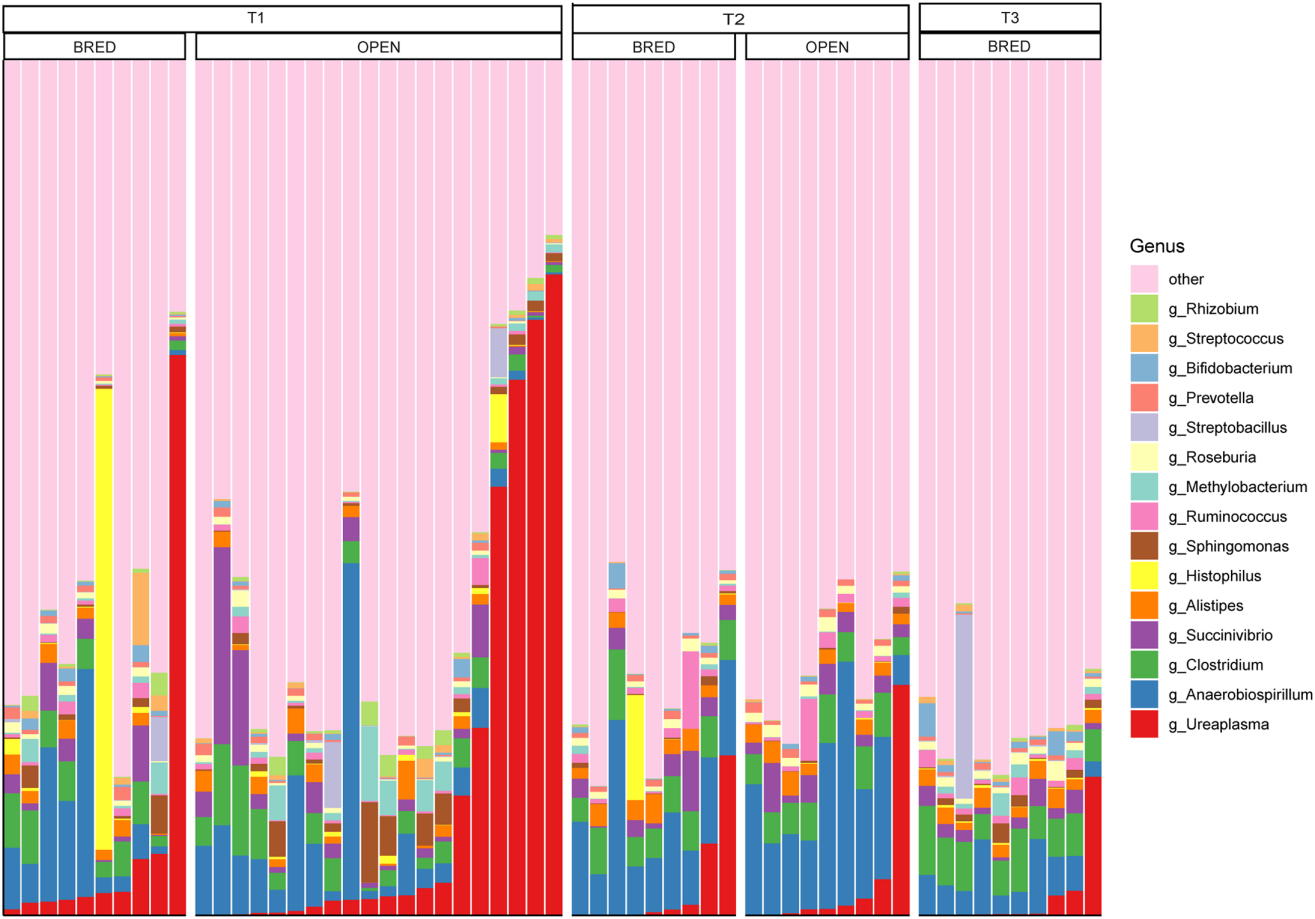
Relative abundance of vaginal microbiota at the genus level.

**Fig. 6:**
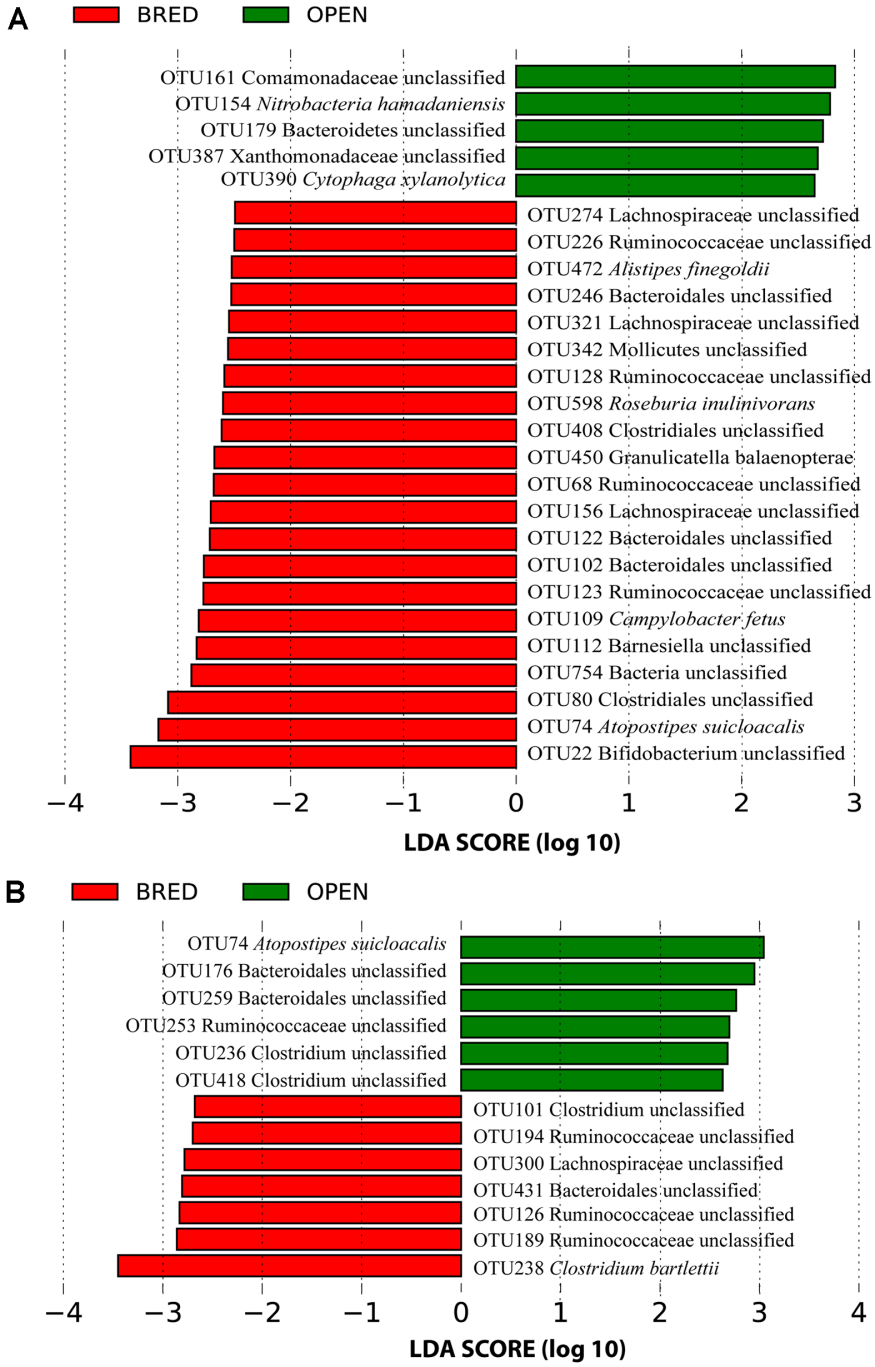
The differentially enriched OTUs in pregnant (BRED) and nonpregnant (OPEN) groups at the first (A)and second (B) AI services.

According to taxonomical annotation, we found that the most common bacterial phyla of vaginal microbiota in dairy cows were *Firmicutes* (with average relative abundance of 32.4%), *Proteobacteria* (19.3%), *Bacteroidetes* (16.2%), *Actinobacteria* (12.5%), and *Tenericutes* (9.0%), respectively (Fig. 4). At the genus level, the most abundant genus was *Ureaplasma* with an average relative abundance of 8.5%, which was followed by *Anaerobiospirillum* (7.9%), *Clostridium* (4.0%) and *Succinivibrio* (2.5%), respectively (Fig. 5). Two strategies were employed for the LEfSe analyses. First, we restrictively analyzed the successfully annotated species and did not detect any statistical difference between BRED and OPEN groups at both the first (T1) and second (T2) AI services. Second, all OTUs with the relative abundance of > 0.1% in more than three samples were subjected to LEfSe analysis. A total of five and 21 OTUs were enriched in BRED and OPEN groups at the T1, respectively (Fig. 6A). At T2, seven OTUs were significantly more abundant in BRED group and six OTUs in OPEN group (Fig. 6B). However, none of these differentially enriched OTUs was overlapped between T1 and T2.

## Discussion

During the past decades, the demographic, behavioral and clinical determinants of vaginal microbiota have been extensively studied for distinguishing the normal and abnormal compositions in humans [22, 23]. Compositional alterations of human vaginal microbiota have been obviously associated with the increased risk of papillomavirus infection and development of cervical cancer [24]. In addition to occurrences of various diseases, vaginal homeostasis is also vital for maintaining healthy physiological functions [25]. In humans, abnormal vaginal microbiota have been found to influence the success rate of in vitro fertilization treatment [26]. In the present study, we accordingly investigated the association of vaginal microbiota composition with pregnancy outcomes of AI service in dairy cows using high-throughput sequencing of 16S rRNA gene.

A similar and recently published study systematically investigated the vaginal and uterine microbiota composition at 21, 9, and 2 days prior to AI service in beef cows and found that the nonpregnant and pregnant cows could be clustered separately according to the uterine microbial profiling only at day 2 [14]. However, a similar clustering pattern between pregnant and nonpregnant cows was inconsistently observed at day 21 for the vaginal microbiota. Deng *et al*. (2019) recently investigated the vaginal and fecal microbiota in beef cows and found significant differences in the diversity of vaginal microbiota between gestation stages [15]. In contrast to the serial samplings before and after AI service in the two similar studies [14, 15], we mainly focused on the compositional characterization of vaginal microbiota at the time of AI service that would be associated with the differential pregnancy outcomes in dairy cows in the present study. We did not further profile vaginal microbiota for the nonpregnant samples at the third AI service because the cows that fail to conceive with more than three AI services will always be culled in practice. Although statistically significant differences were observed among multiple AI services with respect to the alpha diversity of vaginal microbiota, the pregnant and nonpregnant groups could not be distinguished at the first two AI services according to both alpha diversity and beta diversity. Even if a few dozen of OTUs had been identified to be statistically differentially enriched in pregnant and nonpregnant groups, we should be very cautious in drawing such a conclusion because none of them was overlapped between the first and second AI services.

The vaginal tract of mammals provides an ecological niche for a large number of microbial species. In humans, the most predominant bacterial phyla were reported to be the *Firmicutes*, *Bacteroidetes*, *Actinobacteria* and *Fusobacteria*, respectively [27]. However, we found in cows that the most abundant bacterial phyla were *Firmicutes*, *Proteobacteria*, *Bacteroidetes*, and *Actinobacteria*, respectively. These results would suggest inter-species differences in the prevalent microbial species that are colonized in the vaginal tract. At the genus level, we unexpectedly found that the most abundant genus was *Ureaplasma*, which has been associated with various reproductive problems in cattle [28]. Therefore, we speculated that the cows involved in the present study would have subclinical vaginitis because we carefully enrolled only clinically healthy individuals. However, the estimated abundances of *Ureaplasma* genus could not be used for statistically significantly distinguishing the pregnant and nonpregnant cows.
